# Malignant Eyelid Tumors in Italy (2020–2024): Toward Personalized Epidemiologic Insights from Two Referral Centers

**DOI:** 10.3390/jpm15120590

**Published:** 2025-12-02

**Authors:** Lina Corgiolu, Luca Pilloni, Alessandra Di Maria, Maria Angela Romeo, Alessandro Gaeta, Giuseppe Giannaccare, Alberto Cuccu

**Affiliations:** 1 Eye Clinic, Department of Surgical Sciences, University of Cagliari, 09124 Cagliari, Italy; linacorgiolu@gmail.com (L.C.); cuccualberto@yahoo.it (A.C.); 2 Section of Pathology, Department of Medical Science and Public Health, University of Cagliari, 09124 Cagliari, Italy; lucpilloni@tiscali.it; 3 Department of Ophthalmology, Humanitas Research Hospital—IRCCS, 20089 Rozzano, Italy; alessandra.di_maria@humanitas.it; 4 Department of Ophthalmology, University Magna Graecia of Catanzaro, 88100 Catanzaro, Italy; oculistica.romeo.ma@gmail.com; 5 Department of Internal Medicine and Medical Specialties (DIMI), Università Di Genova, 16132 Genova, Italy; alessandrogaeta01@gmail.com

**Keywords:** eyelid malignancies, basal cell carcinoma, squamous cell carcinoma, sebaceous gland carcinoma, epidemiology, Italy, personalized medicine, precision prevention, risk stratification

## Abstract

**Background/Objectives:** Eyelid malignancies represent a clinically relevant subset of cutaneous tumors of the head and neck, with significant functional and cosmetic implications. While basal cell carcinoma (BCC) is the predominant subtype, geographic differences in the relative frequency of squamous cell carcinoma (SCC) and rarer histotypes have been reported. This study aimed at comparing the distribution of malignant eyelid tumors diagnosed in two Italian referral centers, namely Cagliari (Sardinia) and Milan (Lombardy) between 2020 and 2024, and to explore demographic and epidemiologic correlates. **Methods**: A total of 250 malignant eyelid tumors were analyzed: 130 from Cagliari and 120 from Milan. BCC was the most common histological subtype overall (83.2%), followed by SCC (12.4%) and other malignancies (4.4%). The proportion of SCC was significantly higher in Milan (18.3%) compared to Cagliari (6.9%, *p* = 0.04). Logistic regression confirmed Milan as an independent risk factor for SCC (OR 3.79; 95% CI 1.57–9.18; *p* = 0.003). Male gender also emerged as a predictor of SCC (OR 2.50; 95% CI 1.10–5.67; *p* = 0.029). Most cases occurred in patients ≥70 years; cases under 50 years were rare (≈3%). **Conclusions**: BCC remains the predominant malignant eyelid tumor in Italy; significant inter-regional variability exists, with a higher proportion of SCC in northern Italy. These findings highlight the role of environmental, demographic, and organizational factors, and emphasize the need for multicenter registries. Region-specific insights may inform personalized prevention and surveillance strategies for eyelid malignancies. These findings may support the development of region-tailored prevention models and contribute to the growing field of personalized oncology within ophthalmology.

## 1. Introduction

Non-melanoma skin cancers (NMSCs) represent the most frequently diagnosed malignant neoplasms worldwide and constitute a growing public health concern [1]. Within this group, basal cell carcinoma (BCC) is by far the most common cutaneous malignancy in Caucasian populations [2,3]. The estimated life to event risk of developing at least one lesion reaches approximately 30%, a figure that underscores the magnitude of the problem. Over recent decades, the incidence of BCC has steadily increased across Europe. Data from national cancer registries highlight an especially pronounced rise in older age groups and a clear predominance in males [4]. Alongside BCC, cutaneous squamous cell carcinoma (SCC) has shown a progressive upward trend, now accounting for about 20% of keratinocyte carcinomas and carrying a higher risk of metastasis and worse clinical outcomes. Major risk factors include fair skin phenotype, chronic exposure to ultraviolet (UV) radiation, immunosuppression, and a history of previous skin cancers [4,5]. Eyelid tumors show different biological behavior: while BCC usually exhibits indolent growth with a very low metastatic potential (<1%), whereas SCC and especially SGC exhibit more aggressive local invasion and a greater tendency to recur or metastasize [5,6,7,8,9]. Eyelid melanoma, although rare and accounting for fewer than 1% of all cutaneous melanomas, carries a disproportionately high mortality rate and therefore requires specialized diagnostic and therapeutic approaches [8]. Similarly, Merkel cell carcinoma (MCC), another rare eyelid tumor, is notorious for its aggressive course and poor prognosis, underscoring the importance of timely recognition [10].

Eyelid malignancies form a distinct subgroup within cutaneous tumors of the head and neck region [6]. Their impact extends beyond oncologic control, as they often affect a highly visible anatomical site, influencing both functional outcomes (such as eyelid protection and tear film stability) and cosmetic results, with significant implications for patients’ quality of life [11,12]. It is estimated that between 5% and 10% of all cutaneous malignancies occur on the eyelids, and within this group, non-melanoma epithelial tumors are by far the most common [7,13].

Among eyelid NMSCs, BCC typically accounts for 75–85% of cases in Europe and the United States, while SCC represents 7–16%, and sebaceous gland carcinoma (SGC) generally contributes to less than 5% of diagnoses [7,13,14]. Nevertheless, important geographic variations are observed. In Italy and most European countries, BCC clearly predominates [15]. By contrast, in several Asian populations, SGC plays a much more prominent role, reaching proportions of 40–50% of eyelid malignancies in some series. Such differences highlight the interplay of genetic, environmental, and lifestyle factors across populations [14,16].

Against this background, the present study was designed to provide a detailed comparison of malignant eyelid tumors diagnosed and treated at two Italian referral centers, the University Hospital of Cagliari (Sardinia) and the Humanitas Research Hospital in Milan (Lombardy), over a 5-year period (2020–2024). The analysis focuses on histological distribution as well as demographic characteristics of the studied populations, with the aim of contributing to a more comprehensive understanding of the epidemiology of eyelid malignancies in Italy and of exploring potential inter-regional differences. By comparing two Italian regions with contrasting environmental exposures and population characteristics, this study seeks to identify geographic patterns that may inform personalized prevention and surveillance strategies for eyelid malignancies. In the broader context of personalized medicine, integrating epidemiologic, environmental, and demographic data can help refine risk stratification and guide individualized prevention and early detection programs.

## 2. Materials and Methods

This was a retrospective, multicenter observational study carried out at two Italian referral centers: the Eye Clinic of the University Hospital of Cagliari, which serves as the main tertiary ophthalmology hub for southern Sardinia, and the Humanitas Research Hospital in Milan, a high-volume center in northern Italy. Their inclusion ensured broad geographic representation and standardized histopathologic evaluation under comparable national guidelines. Moreover, these centers are situated in regions with distinct environmental exposures and partially different genetic backgrounds, providing an opportunity to explore potential inter-regional variability in eyelid malignancies.

**Study population.** The study included all consecutive histologically confirmed cutaneous eyelid malignancies excised between January 2020 and December 2024.

**Inclusion and exclusion criteria.**

Inclusion criteria: (i) Histologically confirmed diagnosis of a malignant cutaneous eyelid tumor (primary or recurrent); (ii) availability of complete demographic and histopathological data.

Exclusion criteria: (i) Benign lesions; (ii) borderline entities; (iii) incomplete clinical or histopathological data.

Primary and histologically documented recurrent lesions were eligible. Distinct lesions occurring in the same patient (bilateral/synchronous or metachronous) were counted as separate cases. Repeat pathology from the same surgical episode (e.g., margin re-excision of the index lesion) was not double counted. Non-cutaneous primaries (e.g., conjunctival, lacrimal) and borderline lesions were excluded. One eyelid metastasis from breast carcinoma met criteria and was included under “Other malignancies” category.

**Pathology and case ascertainment**. Histopathological assessment was performed independently in the two certified pathology laboratories using harmonized diagnostic protocols and comparable immunohistochemical (IHC) panels when required. For difficult cases, joint review by senior pathologists was undertaken. Classification followed the current WHO criteria for skin tumors; terminology was standardized, and ICD-O-3 topography/morphology codes were mapped where applicable (codes available in the dataset and upon request).

Typical IHC panels included, when indicated: for sebaceous gland carcinoma (SGC), EMA, androgen receptor, adipophilin; for Merkel cell carcinoma (MCC), CK20, synaptophysin/chromogranin, and MCPyV large-T antigen.

For clarity, the “Other malignancies” in the Milan cohort comprised exactly six lesions: 1 SGC, 1 MCC, 2 melanoma in situ, 1 adnexal carcinoma, and 1 eyelid metastasis from breast carcinoma (n = 6).

**Variables and data collection.** For each patient, the following information were systematically collected: Demographic variables (age at diagnosis, gender, and place of residence), histological diagnosis (tumor subtype as determined by standardized histopathological examination performed in the pathology laboratories of the respective centers according to current WHO criteria). Continuous variables (e.g., age) were expressed as mean, standard deviation, and range, while categorical variables (e.g., gender, histological subtype) were reported as absolute frequencies and percentages.

**Ethical Approval and Informed Consent**. The study was conducted in accordance with the Declaration of Helsinki and approved by the Ethics Committee of Humanitas Research Hospital, Milan (protocol code 4967, project “Neoformazioni orbitarie,” approval date 15 July 2025). Given the retrospective design and use of anonymized archival data, the requirement for individual informed consent was formally waived by the Ethics Committee. The institutional protocols of both centers ensure secure data handling and patient confidentiality in compliance with the General Data Protection Regulation (GDPR, EU 2016/679).

**Statistical analysis.** Comparisons between the two centers were conducted to explore potential differences in histological distribution, gender distribution, and age groups. For this purpose, the chi-square test (χ^2^) or Fisher’s exact test (when expected frequencies were small) were applied and proportions were reported with 95% CIs (Wilson method).

The primary regression model was a binary logistic regression with outcome SCC vs. BCC; predictors: center (Milan = 1, Cagliari = 0), gender (male = 1, female = 0), and age (years, continuous). Multicollinearity was assessed (variance inflation factors <2 for all predictors) and model fit (Hosmer–Lemeshow goodness-of-fit). Adjusted odds ratios (aORs) with 95% CIs were calculated. 3; events-per-variable was ≥10 (31 SCC events/3 predictors). Two-sided α = 0.05. All statistical analyses were initially conducted in Excel and subsequently cross-validated using IBM SPSS Statistics, Version 30.0.0 (IBM Corp., Armonk, NY, USA) to ensure robustness and reproducibility of results.

## 3. Results

A total of 250 malignant cutaneous eyelid tumors were included in the study: 130 cases from the center in Cagliari (Sardinia) and 120 from the center in Milan (Lombardy). When considering the overall distribution, BCC clearly emerged as the predominant histological subtype, accounting for more than 4 out of 5 diagnoses, followed by SCC, while SGC, melanoma, and MCC represented only a minority of cases. The complete anonymized dataset, including all demographic, clinical, and histopathological data, is provided in the Supplementary Materials (Table S1).

### 3.1. Histological Distribution

Histopathological subtypes were systematically collected for all cases and are summarized in Table 1 and in the Supplementary Materials (Table S1). In Cagliari, most tumors were BCCs (116 cases, 89.2%), with only 9 SCCs (6.9 Among the remaining malignancies (5 cases, 3.9%), there were 3 sebaceous gland carcinomas (2.3%), 1 Merkel cell carcinoma (0.8%), and 1 melanoma (0.8%). The distribution in Milan was more heterogeneous: although BCCs still predominated (92 cases, 76.7%) and the proportion of SCCs was substantially higher (22 cases, 18.3%). The other malignant tumors (6 cases, 5.0%) included 1 sebaceous gland carcinoma (0.8%), 1 Merkel cell carcinoma (0.8%), 2 melanoma in situ (1.7%), 1 adnexal tumor (0.8%), and 1 case of eyelid localization of breast carcinoma metastasis in a female patient (0.8%), a particularly unusual presentation. Totals refer to lesions rather than unique patients. Recurrent lesions were few (Cagliari: 3; Milan: 4) and are reported within center totals.

**Figure 1:**
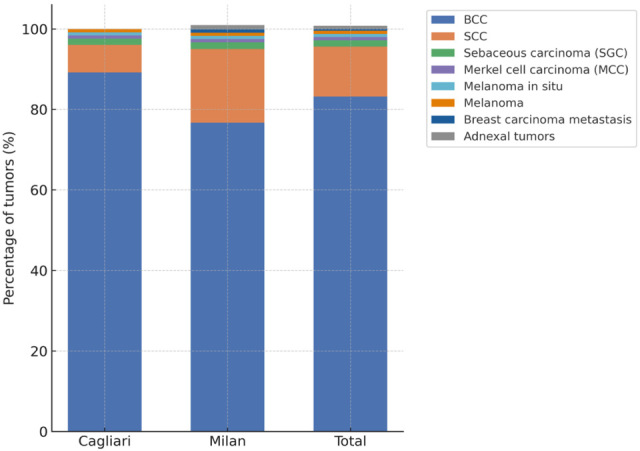
Relative distribution of malignant eyelid tumors by histological subtype in Cagliari and Milan, and in the overall series. Basal cell carcinoma (BCC) was the most common diagnosis in both centers, although squamous cell carcinoma (SCC) was proportionally higher in Milan. Less frequent histotypes included sebaceous gland carcinoma (SGC), melanoma, melanoma in situ, Merkel cell carcinoma (MCC), adnexal tumors, and one case of eyelid localization of breast carcinoma metastasis.

To complement these data, the relative distribution of histological subtypes is also shown graphically (Figure 1), allowing immediate comparison between the two centers.

When pooling the two cohorts, BCC represented 83.2% of all malignant eyelid tumors, SCC accounted for 12.4%, and the remaining 4.4% were distributed among rare histotypes. The difference in the relative frequency of SCC between the two centers was statistically significant (18.3% in Milan vs. 6.9% in Cagliari; χ^2^/Fisher’s exact test, *p* = 0.04), highlighting a key inter-center variation. Tumor recurrence was documented in a small subset of patients. In Cagliari, 3 recurrent cases were identified (2 BCCs and 1 SCC), while in Milan 4 recurrences were observed (3 BCCs and 1 SCC). In line with the study design, all recurrent tumors were included in the overall analysis. A detailed breakdown of histopathological subtypes across the two referral centers is presented in the Supplementary Materials (Table S2).

### 3.2. Demographic Characteristics

Demographic features of the patients are presented in Table 2. Age analysis revealed a marked predominance of elderly patients. In Cagliari, nearly two-thirds of cases (64.6%) occurred in individuals aged ≥70 years, while 32.3% were diagnosed between ages 50–69. Similarly, in Milan, 72.5% of patients were ≥70 years old and 24.2% were aged 50–69. In both cohorts, only about 3% of cases occurred in individuals under 50 years, underscoring the rarity of malignant eyelid tumors in younger patients.

Gender distribution was relatively balanced, though with slight center-specific differences. In Cagliari, cases were slightly more frequent in males (53.8%) than females (46.2%), while in Milan, females were more represented (59.2%) than males (40.8%). These differences did not reach statistical significance (χ^2^ test, *p* = 0.12), but they suggest possible variability in the gender distribution of patients accessing the two referral centers.

### 3.3. Comparative and Regression Analysis

The comparative evaluation between the two centers demonstrated that the proportion of SCC was significantly higher in Milan than in Cagliari (*p* = 0.04). No statistically significant differences were observed for the distribution of BCC, rare histotypes, or gender. The age-related trend confirmed the expected pattern: the incidence of eyelid malignancies progressively increased in older cohorts. The results of the chi-square analyses assessing associations between clinical and pathological variables are presented in the Supplementary Materials (Table S3).

The results of the logistic regression model are summarized in Table 3. Patients treated in Milan had an almost 4-fold higher risk of being diagnosed with SCC compared with those treated in Cagliari (OR 3.79; 95% CI 1.57–9.18; *p* = 0.003). In addition, male gender emerged as an independent risk factor for SCC (OR 2.50; 95% CI 1.10–5.67; *p* = 0.029), while age showed only a borderline trend towards statistical significance (*p* ≈ 0.06), suggesting that its effect may become more evident in larger cohorts. Multivariate regression models evaluating predictors of malignancy and recurrence are summarized in the Supplementary Materials (Table S4).

## 4. Discussion

The results of this study confirm that BCC is the most common malignant eyelid tumor, followed by SCC. However, a significant difference in tumor distribution was observed between the two centers, with SCC being more frequently diagnosed in Milan compared to Cagliari. This finding deserves careful interpretation in the context of national and international literature. Regional variability in environmental exposure, particularly differences in ultraviolet radiation and air pollution between northern and southern Italy, may also contribute to the observed heterogeneity and will be discussed in detail below.

In Italy, data from the Italian Association of Cancer Registries (AIRTUM) indicate an increase in the incidence of non, melanoma skin cancers, although BCC is often underreported [17]. European epidemiologic studies confirm the predominance of BCC (80–90%) and an SCC rate of around 7–15% [18,19]. The German analysis by Warnig et al. [20], the Irish report by Quigley et al. [21], and data from Finland [22] and Bulgaria [23] support these proportions. The Milan cohort (76.7% BCC, 18.3% SCC) falls within the higher limit of SCC prevalence, suggesting possible differences related to environmental and demographic factors, while the Sardinian cohort (89.2% BCC, 6.9% SCC) lies at the lower limit of the European range, with a clear predominance of BCC and a limited share of SCC. It remains to be determined whether this pattern reflects a higher incidence of SCC in Lombardy or, conversely, a relatively lower frequency in Sardinia. In other words, it is unclear whether Milan shows an excess of SCC or Sardinia exhibits a relative deficit compared with European trends. Clarifying this discrepancy is crucial and requires further epidemiologic, demographic, and environmental investigation. To date, no large-scale Italian epidemiologic study has systematically investigated these inter-regional determinants of periocular skin cancer.

### 4.1. Contextualization with Large Registries and Cohorts

These results align with large-scale European series. In Germany, an analysis of over 40,000 adult eyelid neoplasms (2009–2015) confirmed the predominance of BCC (≈85%) and a smaller but increasing proportion of SCC (≈10%) with age [20]. In Ireland, standardized incidence rates showed BCC largely predominant (≈80–85%) and SCC less common (10–12%), with increases in older cohorts [21]. Data from Eastern Europe (Bulgaria) reported 89.6% BCC and 7.2% SCC, while in Finland eyelid SCC had a standardized incidence of ≈1 per 100,000 inhabitants, with a median age at diagnosis of 79 years [22,23]. Outside Europe, large hospital-based cohorts from southern China (>5000 lesions) documented a notable burden of SC (12%) and SCC (up to 30%), with BCC remaining the most common [24], underscoring the geographic variability already discussed.

### 4.2. Gender Disparities and Age Groups

A recent systematic review on head and neck SCC reported higher risk in males and, in some series, less favorable outcomes compared with females [25]. Other European studies confirmed both higher incidence and more aggressive clinical courses in males [21,23]. This is consistent with this multivariate analysis, in which the male gender emerged as an independent factor associated with SCC diagnosis (OR 2.50). Possible explanations include greater cumulative UV exposure [20], lifestyle factors such as outdoor work and potentially smoking habits, although the evidence for a direct causal role of smoking in skin carcinogenesis remains limited [5]. Lower utilization of screening or routine dermatologic assessments has also been reported in males [26]. Although most diagnoses occur in patients ≥70 years, the literature also describes presentations in younger individuals, sometimes associated with genetic syndromes or immunodeficiency conditions; diagnostic delays and atypical histologic patterns have been reported [27]. In this sample, cases < 50 years represented approximately 3% in both centers, consistent with the rarity of these forms.

### 4.3. Minority Cases

The present series also included one case of eyelid metastasis from breast carcinoma, a rare occurrence but described in the literature as the most common type of eyelid metastasis [28,29,30,31]. Sebaceous carcinoma, eyelid melanoma, and Merkel cell carcinoma were only rarely observed in the present cohort but warrant attention due to their aggressiveness and unfavorable prognosis, as highlighted in large international series [32,33,34]. In particular, sebaceous carcinoma is frequently underdiagnosed and can mimic benign lesions [35,36], whereas eyelid melanoma and MCC, though rare, have high recurrence and mortality rates [37,38].

### 4.4. Interpretation of Regional Differences

Understanding the possible causes of the frequency difference between the Cagliari and Milan centers is of particular interest. Geographically, Cagliari and Milan are located approximately 700 km apart (39° N vs. 45° N latitude), corresponding to markedly different climatic conditions across the Italian peninsula. The higher frequency of SCC in Milan may be related to several factors.

**Environmental aspects** likely play a central role, with markedly different exposures between regions: Lombardy is characterized by some of the highest concentrations of particulate matter and ozone in Italy, whereas Sardinia shows one of the highest annual solar UV indices in Europe but minimal industrial pollution [39,40].

**Demographics** could be another explanation, with an older average age in the Lombardy population, or organizational and referral-related, as high-volume centers like Milan are more likely to diagnose and manage complex cases [41].

**Genetic background** could also contribute, as population isolates such as Sardinia are known to exhibit distinctive genetic structures within Italy [42]. These genetic determinants may interact with environmental exposures, amplifying or mitigating local risk profiles and ultimately shaping individualized susceptibility patterns that are central to personalized medicine. As a matter of fact, some authors emphasize the role of genetic variants related to skin pigmentation and DNA repair capacity, which may explain differential susceptibility to BCC, SCC, or SC in various populations [16,43].

**Ultraviolet radiation** is a well-established driver of keratinocyte carcinogenesis, with cumulative exposure identified as a key driver in development SCC in equatorial and subtropical regions [24,44]. According to national meteorological and UV-monitoring data, the average annual sunlight exposure is about 2600 h in Cagliari and 1800 h in Milan [39,40].

**Urban air pollution** has been associated with elevated NMSC risk through mechanisms involving oxidative stress, inflammation and impaired cutaneous immunity [45,46]. Fine particulate matter (PM2.5 and PM10), polycyclic aromatic hydrocarbons, nitrogen oxides, and ground-level ozone can penetrate the cutaneous barrier and generate reactive oxygen species (ROS), leading to oxidative DNA damage in keratinocytes. Chronic exposure induces the activation of pro-inflammatory signaling cascades, including NF-κB and the aryl hydrocarbon receptor (AhR), while also interacting with the Nrf2/NQO1 pathway, a key regulator of oxidative stress responses. These alterations promote chronic inflammation and impair local immune surveillance, ultimately facilitating mutagenesis and malignant transformation of epidermal cells [47,48].

Recent reviews highlight that these mechanisms may synergize with ultraviolet radiation, amplifying carcinogenic risk in polluted environments with high levels of particulate pollutants [49,50]. Lifestyle factors, such as outdoor occupations, sun protection practices, and socioeconomic differences, also play a significant role in shaping local epidemiology [5,26,51].

Taken together, these epidemiologic patterns suggest that the interplay between environmental exposure, genetic susceptibility, and regional demographics likely underlies the observed variability in SCC/BCC ratios between northern and southern Italy and deserve further investigation in future multicenter studies.

### 4.5. International Comparison

Comparable geographic variations have been documented globally, further supporting the influence of genetic and environmental factors on tumor distribution. In Asia, and particularly in India [52], SGC is much more frequent, accounting for up to 40% of diagnoses in some series [16,43,53]. In China, large cohorts (>5000 cases analyzed) confirm a relevant proportion of SGC (≈12%) and SCC (up to 30%), alongside a still predominant but comparatively lower proportion of BCC [24]. In Brazil, a multicenter series reported approximately 61% BCC, 30% SCC, and a small proportion of SGC, placing the region at higher SCC frequencies than Europe [45]. Similarly, in tropical and high-UV exposure regions such as Australia, where annual sunlight exceeds 2800–3000 h, SCC accounts for approximately 25–30% of eyelid malignancies—among the highest proportions worldwide—reflecting the strong association between cumulative ultraviolet radiation and keratinocyte carcinogenesis [25]. These global findings support the influence of geographic, genetic, and environmental determinants in shaping tumor distribution, consistent with the regional contrasts observed between northern and southern Italy.

### 4.6. Study Limitations

The present analysis has several limitations that should be acknowledged, underlining its exploratory nature. It should be interpreted as a hypothesis-generating comparison aimed at informing the design of prospective multicenter registries with harmonized clinical, surgical, and follow-up data.

First, its retrospective and pathology-based design entails the inherent constraints of data completeness and potential referral bias, as both centers are tertiary institutions that may not fully reflect population-level trends. Moreover, detailed clinical information such as tumor size, precise eyelid location, or follow-up outcomes was not available; therefore, classification of lesions according to the current TNM system was not possible. The absence of denominator or incidence data also limits direct epidemiologic comparisons. Another limitation is the potential referral bias, linked to the organizational and demographic characteristics of the two centers, which may have influenced case distribution. In addition, lifestyle and behavioral variables that may influence eyelid cancer risk—such as the use of spectacles, occupational or recreational sun exposure, and other environmental or personal habits—were not systematically collected. The lack of data prevents adjustment for individual-level protective or risk factors and represents an important limitation for interpreting regional differences. Finally, the relatively small number of SCC cases may affect the robustness of regression analyses.

### 4.7. Future Perspectives

Looking ahead, prospective multicenter studies involving larger and more diverse patient populations are needed to provide more robust and generalizable data. Establishing comprehensive and regularly updated national registries is essential to capture not only BCC cases but also rarer forms. In this context, dataset standardization (histotype according to current classifications, tumor size, precise eyelid location, surgical margins, perineural/lymphovascular invasion, surgical technique, standard excision vs. Mohs, and reconstruction modality) is a prerequisite for reliable inter-center comparisons. Future multicenter prospective studies with harmonized follow-up schedules are needed to assess recurrence and metastasis more accurately, allowing better benchmarking with international datasets.

At the same time, future research should investigate the role of environmental factors, such as UV exposure and air pollution, using objective metrics (cumulative local UV dose, annual averages of PM2.5/PM10 and photochemical oxidants), integrating these with individual characteristics (skin phototype, outdoor activity, immunosuppression). The analysis of molecular pathways (NRF2/NQO1, aryl hydrocarbon receptor—AhR) may help elucidate pathogenetic mechanisms and identify prognostic biomarkers useful for risk stratification [54,55]. Finally, time-to-event outcome studies with structured follow-up (e.g., quarterly visits in the first year, semiannual up to 3 years, then annual) would help define high-yield surveillance windows and measure recurrence and metastasis more precisely by histotype and surgical technique.

The regional variability observed in this study highlights the importance of adopting a personalized approach to prevention and management of eyelid malignancies. Integrating clinical, demographic, environmental, and—where available—molecular data can enable refined risk stratification and tailored follow-up schedules. The identification of environmental and genetic determinants may contribute to precision public health strategies, optimizing surveillance intensity according to individual and regional risk profiles. This approach is aligned with the principles of personalized medicine, aiming to translate population-level evidence into patient- and region-specific care pathways.

## 5. Conclusions

In summary, this multicenter analysis confirm that BCC is the most frequent malignant eyelid tumor in the Italian population, while SCC shows a less uniform distribution, with significantly higher prevalence in Milan compared with Cagliari. This difference, consistent with some European series but contrasting with the more homogeneous trends reported elsewhere, may be explained by a combination of environmental factors, such as the severe air pollution typical of the Po Valley [47,48] or the higher UV exposure in southern and insular regions such as Sardinia [39,40], along with demographic and organizational differences between the two cohorts. Previous evidence indicates that chronic exposure to air pollution, particularly fine particulate matter and ozone, can increase the risk of cutaneous squamous cell carcinoma through oxidative and inflammatory mechanisms. In Italy, marked regional contrasts exist in environmental conditions, with higher particulate concentrations in Lombardy and greater annual solar UV exposure in Sardinia. These factors may act synergistically, modulating carcinogenic risk.

Overall, this multicenter comparison provides new evidence that environmental and demographic contrasts between northern and southern Italy may partly account for the observed inter-regional variability in eyelid tumor distribution, underscoring the need for region-tailored preventive strategies. Targeted preventive strategies, such as photoprotection in elderly and outdoor workers, patient education on eyelid self-examination, and early diagnosis programs in high-burden areas, especially for more aggressive subtypes (SCC, SC, MCC). Equally important is the implementation of standardized clinical pathways with margin-controlled approaches (Mohs/staged excisions) for anatomically critical sites, together with structured follow-up protocols to ensure early detection of recurrence and metastasis.

Looking ahead, an integrated research infrastructure combining clinical registries, environmental data, and molecular information will enhance the understanding of risk determinants and guide more equitable and effective health policies and therapeutic decisions. In this sense, the study contributes to the foundation of personalized ophthalmic oncology, supporting the transition from population-based statistics toward individualized prevention, diagnosis, and surveillance models.

## Figures and Tables

**Table 1:** Histological distribution of malignant eyelid tumors.

Center	BCC n (%)	SCC n (%)	Others n (%)	Total
Cagliari	116 (89.2)	9 (6.9)	5 (3.9)	130
Milan	92 (76.7)	22 (18.3)	6 (5.0) ^1^	120
Total	208 (83.2)	31 (12.4)	11 (4.4)	250

^1^ Note: Milan includes one case of eyelid localization of breast carcinoma metastasis in a female patient.

**Table 2:** Demographic characteristics of patients with malignant eyelid tumors.

Center	Age <50 n (%)	Age 50–69 n (%)	Age ≥70 n (%)	Female n (%)
Cagliari	4 (3.1)	42 (32.3)	84 (64.6)	60 (46.2)
Milan	4 (3.3)	29 (24.2)	87 (72.5)	71 (59.2)

**Table 3:** Comparative analysis of SCC vs. BCC.

Variable	OR	95% CI	*p*-Value
Milan vs. Cagliari	3.79	1.57–9.18	0.003
Male gender	2.50	1.10–5.67	0.029
Age (per year)	1.04	0.99–1.08	0.06

## Data Availability

Data is contained within the article or Supplementary Materials.

## Supplementary Materials

The following supporting information can be downloaded at: https://www.mdpi.com/article/10.3390/jpm15120590/s1, Table S1: Histological; Table S2: Demographics; Table S3: Chi-square; Table S4: Regression.

## Abbreviations

The following abbreviations are used in this manuscript:

BCC	Basal Cell Carcinoma
SCC	Squamous Cell Carcinoma
SGC	Sebaceous Gland Carcinoma
MCC	Merkel Cell Carcinoma
NMSC	Non-Melanoma Skin Cancer
UV	Ultraviolet Radiation
OR	Odds Ratio
CI	Confidence Interval
ROS	Reactive Oxygen Species
AhR	Aryl Hydrocarbon Receptor
Nrf2/NQO1	Nuclear Factor Erythroid 2–Related Factor 2/NAD(P)H Quinone Dehydrogenase 1

## References

[1] Ciuciulete A.-R., Stepan A.E., Andreiana B.C., Simionescu C.E. (2022). Non-Melanoma Skin Cancer: Statistical Associations between Clinical Parameters. Curr. Health Sci. J..

[2] Rojas K.D., Perez M.E., Marchetti M.A., Nichols A.J., Penedo F.J., Jaimes N. (2022). Skin cancer: Primary, secondary, and tertiary prevention. Part II. J. Am. Acad. Dermatol..

[3] Yélamos O., Geller S., Tokez S. (2023). Skin cancer special issue in Skin Health and Disease. Ski. Health Dis..

[4] Peris K., Fargnoli M.C., Kaufmann R., Arenberger P., Bastholt L., Seguin N.B., Bataille V., Brochez L., del Marmol V., Dummer R. (2023). European consensus-based interdisciplinary guideline for diagnosis and treatment of basal cell carcinoma—Update 2023. Eur. J. Cancer.

[5] Stratigos A.J., Garbe C., Dessinioti C., Lebbe C., van Akkooi A., Bataille V., Bastholt L., Dreno B., Dummer R., Fargnoli M.C. (2023). European consensus-based interdisciplinary guideline for invasive cutaneous squamous cell carcinoma. Part 1: Diagnostics and prevention—Update 2023. Eur. J. Cancer.

[6] Moran J.M., Phelps P.O. (2020). Periocular skin cancer: Diagnosis and management. Disease-a-Month.

[7] Mukarram M., Khachemoune A. (2024). Upper and Lower Eyelid Malignancies: Differences in Clinical Presentation, Metastasis, and Treatment. Arch. Dermatol. Res..

[8] Pinna G., Dell’antonia M., Atzori L., Ferreli C., Casula L., Minerba L., Faa G., Rongioletti F., Pilloni L. (2023). Recurrent nasal basal cell carcinoma treated with standard surgery excision: Evaluation of volume ratio. Ital. J. Dermatol. Venereol..

[9] Di Maria A., Barone G., Ferraro V., Tredici C., Manara S., De Carlo C., Gaeta A., Confalonieri F. (2023). Recurrence of Basal Cell Carcinoma Treated with Surgical Excision and Histopathological Analysis with Frozen Section Technique with Complete Margin Control (CMC-FS): A 15-Year Experience of a Reference Center. Cancers.

[10] Robbins J.O., Huck N.A., Khosravi P., Torabi S.J., Woodward J.A., Kuan E.C., Dermarkarian C.R. (2025). Trends in Demographic, Clinical, Socioeconomic, and Facility-Specific Factors Linked to Eyelid Melanoma Survival: A National Cancer Database Analysis. Ophthalmic Plast. Reconstr. Surg..

[11] Romeo M.A., Taloni A., Borselli M., Di Maria A., Mancini A., Mollace V., Carnovale-Scalzo G., Scorcia V., Giannaccare G. (2025). Iatrogenic Ocular Surface Complications After Surgery for Ocular and Adnexal Tumors. Cancers.

[12] Di Maria A., Barone G., Gaeta A., Confalonieri F., Vinciguerra P., Vinci V., Klinger M., Ferraro V. (2024). Persistent Conjunctival Chemosis after Lower Lid Blepharoplasty: A Comparison of Different Surgical Techniques. J. Clin. Med..

[13] Silverman N., Shinder R. (2017). What’s new in eyelid tumors. Asia-Pac. J. Ophthalmol..

[14] Sato Y., Takahashi S., Toshiyasu T., Tsuji H., Hanai N., Homma A. (2024). Squamous cell carcinoma of the eyelid. Jpn. J. Clin. Oncol..

[15] Sordi E., Piscitelli P., Albanese C., Melcarne A., Tardio A., Quarta F., Greco E., Miani A., Falco A., De Matteis E. (2024). Incidence of Non-Melanoma Skin Cancers in Salento (Southern Italy): A 15-Year Retrospective Analysis from the Cancer Registry of Lecce. Epidemiologia.

[16] Kaliki S., Vempuluru V.S., Tanna V., Luthra A. (2025). Eyelid and periocular sebaceous gland carcinoma: Risk factors for recurrence, exenteration, metastasis, and death in 355 patients. Can. J. Ophthalmol..

[17] AIRTUM Working Group, Rete Tumori Rari (RTR) (2019). Rare Cancers in Italy: Epidemiology and Survival. Epidemiol. Prev..

[18] Alfaar A.S., Suckert C.N., Rehak M., Girbardt C. (2023). The epidemiology of adults’ eyelid malignancies in Germany between 2009 and 2015; An analysis of 42,710 patients’ data. Eur. J. Ophthalmol..

[19] Galindo-Ferreiro A., Sanchez-Tocino H., Diez-Montero C., Belani-Raju M., García-Sanz R., Diego-Alonso M., Llorente-Gonzalez I., Perez P.C., Ferrer-Gómez A., Sales-Sanz M. (2022). Primary periocular squamous cell carcinoma in central Spain: Factors related to recurrence. Eur. J. Ophthalmol..

[20] Warnig C., Wollina U. (2021). Cutaneous squamous cell carcinoma of head and neck region: A single center analysis of 1296 tumors with clinical characteristics, comorbidities, treatment, and sun-protection behavior. Dermatol. Ther..

[21] Quigley C., Deady S., Hughes E., McElnea E., Zgaga L., Chetty S. (2019). National incidence of eyelid cancer in Ireland (2005–2015). Eye.

[22] Niinimäki P., Siuko M., Tynninen O., Kivelä T.T., Uusitalo M. (2024). Cutaneous squamous cell carcinoma of the eyelid in northern latitudes, a 25-year experience in Finland. Acta Ophthalmol..

[23] Zlatarova Z.I., Dokova K.G. (2021). Incidence of Non-melanoma Eyelid Malignancies in Bulgaria (2000–2015). Ophthalmic Epidemiol..

[24] Wang L., Shan Y., Dai X., You N., Shao J., Pan X., Gao T., Ye J. (2021). Clinicopathological analysis of 5146 eyelid tumours and tumour-like lesions in an eye centre in South China, 2000–2018: A retrospective cohort study. BMJ Open.

[25] Tan B., Seth I., Fischer O., Hewitt L., Melville G., Bulloch G., Ashford B. (2022). Sex Disparity for Patients with Cutaneous Squamous Cell Carcinoma of the Head and Neck: A Systematic Review. Cancers.

[26] Stratigos A.J., Garbe C., Dessinioti C., Lebbe C., van Akkooi A., Bataille V., Bastholt L., Dreno B., Dummer R., Fargnoli M.C. (2023). European consensus-based interdisciplinary guideline for invasive cutaneous squamous cell carcinoma: Part 2. Treatment—Update 2023. Eur. J. Cancer.

[27] Biazim D.F., Osaki M.H., Kikkawa D.O., Liu C.Y., Leonardo F., Osaki T.H. (2022). Eyelid malignancies in young individuals: Clinical peculiarities. Int. Ophthalmol..

[28] De Giorgi V., Venturi F., Portelli F., Maida P., Scarfì F., Trane L., Gori A., Silvestri F., Santoro N., Massi D. (2020). Eyelid skin metastasis as first sign of breast cancer recurrence. Breast J..

[29] Samir Alfaar A., Saad A.M., Khalafallah M.T., Ezzat Elsherif O., Moataz Hamed O., Ola S. (2020). Second primary malignancies of eye and ocular adnexa after a first primary elsewhere in the body. Zenodo.

[30] Sindoni A., Fama’ F., Vinciguerra P., Dionigi G., Manara S.A.A.M., Gaeta R., Gioffre’-Florio M., Di Maria A. (2020). Orbital metastases from breast cancer: A single institution case series. J. Surg. Oncol..

[31] Sindoni A., Di Maria A., Fama’ F. (2020). Orbital metastases in infiltrating lobular carcinoma of the breast. J. Surg. Oncol..

[32] Huck N.A., Khosravi P., Torabi S.J., Zhou P.S., Nguyen T.V., Tao J.P., Kuan E.C., Dermarkarian C.R. (2025). Survival Outcomes of 997 Patients with Eyelid Sebaceous Carcinoma in the National Cancer Database. J. Craniofacial Surg..

[33] Komatsu H., Usui Y., Sukeda A.O.I., Yamamoto Y., Ohno S.I., Goto K., Kuroda M., Nagao T., Goto H. (2023). Prevalence of Merkel Cell Polyomavirus in Primary Eyelid Merkel Cell Carcinomas and Association with Clinicopathological Features. Am. J. Ophthalmol..

[34] Pisano C.E., Trager M.H., Fan W., Samie F.H. (2024). Surgical margins and outcomes for eyelid melanoma: A systematic review and meta-analysis. Arch. Dermatol. Res..

[35] Drozdowski R., Grant-Kels J.M., Falcone M., Stewart C.L. (2024). Adnexal neoplasms of the eye. Clin. Dermatol..

[36] Dowell-Esquivel C., Lee R., DiCaprio R.C., Nouri K. (2024). Sebaceous carcinoma: An updated review of pathogenesis, diagnosis, and treatment options. Arch. Dermatol. Res..

[37] Levinkron O., Schwalb L., Shoufani A., Gutovitz J., Krausz J., Briscoe D. (2024). Comparison of the clinical characteristics of benign and malignant eyelid lesions: An analysis of 1423 eyelid lesions, compared between ophthalmology department and plastics department. Graefe’s Arch. Clin. Exp. Ophthalmol..

[38] Go C.C., Kim D.H., Go B.C., McGeehan B., Briceño C.A. (2022). Clinicopathologic Characteristics and Prognostic Factors Impacting Survival in Melanoma of the Eyelid. Am. J. Ophthalmol..

[39] Vitt R., Laschewski G., Bais A., Diémoz H., Fountoulakis I., Siani A.-M., Matzarakis A. (2020). UV-Index Climatology for Europe Based on Satellite Data. Atmosphere.

[40] Meloni D., Casale G.R., Siani A.M., Palmieri S., Cappellani F. (2007). Solar UV Dose Patterns in Italy. Photochem. Photobiol..

[41] Brodowski R., Pakla P., Dymek M., Migut M., Ambicki M., Stopyra W., Ozga D., Lewandowski B. (2019). Clinical-pathological characteristics of patients treated for cancers of the eyelid skin and periocular areas. Adv. Clin. Exp. Med..

[42] Di Gaetano C., Voglino F., Guarrera S., Fiorito G., Rosa F., Di Blasio A.M., Manzini P., Dianzani I., Betti M., Cusi D. (2012). An Overview of the Genetic Structure within the Italian Population from Genome-Wide Data. PLoS ONE.

[43] Thiagarajan S., Bahani A., Chaukar D., Dcruz A.K. (2020). Eyelid carcinoma: An experience from a tertiary cancer center. J. Cancer Res. Ther..

[44] Silva G.S., Rosenbach M. (2021). Climate change and dermatology: An introduction to a special topic, for this special issue. Int. J. Women’s Dermatol..

[45] Dias M.R., Osaki M.H., Ferreira C.A.A., Conti M.L., Cho S., Nicolau Z., Osaki T.H. (2021). Epidemiological Profile and Clinical Stage at Presentation of Eyelid Malignancies in a Multi-Ethnic Country. J. Craniofacial Surg..

[46] Lehman J.S., Erickson L.A. (2023). Emerging concepts in dermatopathology: A special issue of neoplastic, inflammatory, and special-site dermatopathology and important practice considerations. Hum. Pathol..

[47] RPA Lombardia (2025). Qualità Dell’aria: Un Primo Bilancio Dell’anno 2024.

[48] Hamer P.D., Fjæraa A.M., Colette A., Rouil L., Kuenen J., Mortier A., Tsyro S., Ung A., Sierra N., Pagonabarraga G. (2024). CAMS Assessment Report on European Air Quality 2024.

[49] Gu X., Li Z., Su J. (2024). Air pollution and skin diseases: A comprehensive evaluation of the associated mechanism. Ecotoxicol. Environ. Saf..

[50] Hedderson M.M., Asgari M.M., Xu F., Quesenberry C.P., Sridhar S., Geier J., Lemeshow A.R. (2023). Rates of malignancies among patients with moderate to severe atopic dermatitis: A retrospective cohort study. BMJ Open.

[51] Safi S., Ahmadzade M., Karimi S., Akbari M.E., Rouientan H., Abolhosseini M., Kanavi M.R., Khorrami Z. (2023). A registration trend in eyelid skin cancers and associated risk factors in Iran, 2005–2016. BMC Cancer.

[52] Gupta R., Bhaduri A., Desai S., Das S., Menon V. (2020). Malignant tumors of the eyelid in India: A multicenter, multizone study on clinicopathologic features and outcomes. Indian J. Ophthalmol..

[53] Goto H., Yamakawa N., Komatsu H., Asakage M., Tsubota K., Ueda S., Nemoto R., Shibata M., Umazume K., Usui Y. (2022). Epidemiological characteristics of malignant eyelid tumors at a referral hospital in Japan. Jpn. J. Ophthalmol..

[54] Akhtar S., Hourani S., Therachiyil L., Al-Dhfyan A., Agouni A., Zeidan A., Uddin S., Korashy H.M. (2022). Epigenetic Regulation of Cancer Stem Cells by the Aryl Hydrocarbon Receptor Pathway. Semin. Cancer Biol..

[55] Yang Q., Yan R., Mo Y., Xia H., Deng H., Wang X., Li C., Kato K., Zhang H., Jin T. (2022). The Potential Key Role of the NRF2/NQO1 Pathway in the Health Effects of Arsenic Pollution on SCC. Int. J. Environ. Res. Public Health.

